# Assessment of the diagnostic value of serum cathepsin S and its correlation with HDL subclasses in patients with non-Hodgkin's lymphoma

**DOI:** 10.5937/jomb0-48959

**Published:** 2024-09-06

**Authors:** Bosa Mirjanić-Azarić, Siniša Stanković, Zana Radić-Savić, Dragana Malčić-Zanić, Ana Ninić, Marija Vuković, Lana Nezić, Ranko Skrbić, Nataša Bogavac-Stanojević

**Affiliations:** 1 University of Banja Luka, Faculty of Medicine, Department of Medical Biochemistry, Banja Luka, The Republic of Srpska, Bosnia and Herzegovina; 2 University Clinical Centre of the Republic of Srpska, Institute of Laboratory Diagnostic, Banja Luka, Republic of Srpska, Bosnia and Herzegovina; 3 University Clinical Centre of the Republic of Srpska, Department of Nuclear Medicine, Banja Luka, The Republic of Srpska, Bosnia and Herzegovina; 4 University of Banja Luka, Faculty of Medicine, Banja Luka, Republic of Srpska, Bosnia and Herzegovina; 5 University of Banja Luka, Faculty of Medicine, Department of Pediatrics, Banja Luka, The Republic of Srpska, Bosnia and Herzegovina; 6 University of Belgrade, Faculty of Pharmacy, Department of Medical Biochemistry, Belgrade, Serbia; 7 University of Banja Luka, Faculty of Medicine, Department of Pharmacology, Toxicology and Clinical Pharmacology, Banja Luka, Republic of Srpska, Bosnia and Herzegovina; 8 Academy of Sciences and Arts of the Republic of Srpska, Banja Luka, The Republic of Srpska, Bosnia and Herzegovina

**Keywords:** cathepsin S, cystatin C, HDL3a and HDL3b sub classes, apoptosis, Hodgkin and non-Hodgkin lymphoma, katepsin S, cistatin C, HDL3a i HDL3b subklase, apoptoza, Hodgkinov i ne-Hodgkinov limfom

## Abstract

**Background:**

Recent findings point to the key role of cathepsin S (CTSS) in the survival of malignant cells, as well as the significance of the anti-apoptotic properties of high-density lipoprotein (HDL) that contribute to enhanced cell survival. The purpose of this study is to analyse CTSS as a potential biomarker in lymphoma. Also, in order to better understand the role of CTSS in the origin and development of lymphoma, its association with cystatin C (Cys C), lipids, and inflammatory markers was analysed.

**Methods:**

The study included 90 subjects: 11 Hodgkin (HL) and 44 B-cell non-Hodgkin lymphoma (NHL) patients, as well as 35 healthy subjects. CTSS was determined using the Invitrogen ELISA kit (Thermo Fisher Scientific, Inc., Waltham, MA, USA). The non-denaturing 3%-31% polyacrylamide gradient gel electrophoresis method was used to separate plasma HDL particles.

**Results:**

The level of CTSS was significantly higher in NHL patients than in control subjects: 12.20 (9.75-14.57) vs 9.97 (8.44-10.99), P<0.001. In NHL patients, there was a positive correlation between CTSS and the proportions of HDL3a, HDL3b, and the sum of the HDL3 subclasses (r=0.506, P<0.001; r=0.411, P=0.006, r=0.335, P=0.026, respectively). In addition, the area under the receiver operating characteristic curve (AUC curve) of CTSS was 0.766 (CI: 0.655-0.856) for NHL patients. There was no significant difference in CTSS values between the control group and patients with HL, nor significant correlations between CTSS and HDL subclasses in the HL group.

**Conclusions:**

CTSS is significantly elevated in patients with NHL and has the potential to be a new diagnostic bio - marker for the detection of NHL. Also, this study was the first to unveil the association between serum CTSS levels and the proportions of anti-apoptotic HDL3a and HDL3b subclasses in NHL patients.

## Abstract (sr)

**Uvod:**

Novija otkrića ukazuju na ključnu ulogu katepsina S (CTSS) u preživljavanju malignih ćelija, kao i na značaj anti-apoptotičkih osobina lipoproteina visoke gustoće (HDL) koje doprinose većem preživljavanja ćelija. Cilj ove studije je analizirati CTSS kao potencijalni biomarker kod limfomu. Takođe, kako bi se bolje razumela uloga CTSS-a u nastanku i razvoju limfoma, analizirana je njegova povezanost sa cistatinom C (Cys C), lipidima i upalnim markerima.

**Metode:**

U istraživanje je bilo uključeno 90 ispitanika: 11 bolesnika sa Hodgkinovim (HL) i 44 bolesnika sa B-ćelijskim ne-Hodgkinovim limfomom (NHL), te 35 zdravih ispitanika. Za merenje serumskog CTSS je korišćen Invitrogen ELISA kit (Thermo Fisher Scientific, Inc., Waltham, MA, SAD). HDL subfrakcije su razdvojene metodom vertikalne elektroforeze na gradijentu poliakrilamida.

**Rezultati:**

Nivo CTSS bio je značajno viši kod pacijenata sa NHL-om nego kod ispitanika kontrolne grupe: 12,20 (9,75-14,57) naspram 9,97 (8,44-10,99), P<0,001. Takođe, kod pacijenata s NHL-om postojala je pozitivna korelacija između CTSS i proporcije HDL3a, HDL3b i HDL3 potklasa (r=0,506, P<0,001; r=0,411, P=0,006, r=0,335, P =0,026). Osim toga, površina ispod ROC krive (AUC) CTSS bila je 0,766 (CI: 0,655-0,856) za NHL pacijente. Nije bilo značajne razlike u vrednostima CTSS između kontrolne grupe i pacijenata sa HL-om, kao niti značajnih korelacija između CTSS i HDL podklasa.

**Zaključak:**

CTSS je bio značajno povišen kod pacijenata sa NHL što pokazuje da CTSS ima potencijal novog dijagnostičkog biomarkera za otkrivanje NHL. Takođe, ovo istraživanje je prvo koje je pokazalo da postoji korelacija između serumskog CTSS i udela anti-apoptotskih podklasa HDL3a i HDL3b kod pacijenata s NHL-om.

## Introduction

Lymphomas are widely distributed hematologic malignancies that originate from malignantly transformed B and T lymphocytes, as well as NK cells. According to the World Health Organization classification, lymphomas represent a heterogeneous group with greater than 80 subtypes [1]. These cancers are categorised into two main groups, the most common of which is non-Hodgkin lymphoma (NHL) and the less frequent group of Hodgkin lymphoma (HL). The diagnosis of lymphomas at an initial stage is essential; therefore, looking for a new, good serum marker is important to improve lymphoma diagnosis, monitoring, and treatment [2].

Review articles point out the involvement of cathepsin S (CTSS) in various processes, such astumour microenvironment remodelling, angiogenesis promotion, tumour migration and growth [3] [4]. Given that CTSS released from lysosomes triggers apoptosis by activating pro-apoptotic Bid and degrading anti-apoptotic Bcl-2 family, cathepsins are involved in the control of cell death or survival [3].

CTSS is a lysosomal protease that plays an important role in the catabolism of intracellular proteins, commonly with an activity optimum at an acidic pH [5]. Both acidic tumour microenvironment and glycosaminoglycans accelerate the autocatalytic activation of cathepsins and enhance their stability in the extracellular matrix (ECM) [6]. CTSS plays a key role in cancer progression, angiogenesis, and metastasis, particularly through its ability to degrade the ECM [7]. Altogether, the tumour cells overexpress cysteine cathepsins to increase opportunities for survival, proliferation, motility, and invasion. Recent studies have shown that inhibition of CTSS reduces angiogenesis, increases apoptosis, and reduces tumour volume and invasion. Therefore, CTSS could be a viable target for cancer treatment [8].

Besides that, recent evidence suggests that during lymphomagenesis, a decrease in circulating high-density lipoprotein cholesterol (HDL-C) may exist [9] [10]. However, some studies indicated that CTSS could potentially affect not only HDL but also low-density lipoprotein (LDL) [11]. The cathepsin-secreting cells induce rapid depletion of lipid-poor (pre-beta-HDL) and lipid-free apoA-I while, on the other hand, inhibiting cellular cholesterol efflux [12]. Namely, the transport of HDL-mediated cholesterol is closely associated with malignant cell survival and tumour development [13]. In that way, previous studies demonstrated that HDL particles stimulated the growth and proliferation of breast carcinoma, primarily HDL3 particles [13] [14]. Also, the knowledge about the anti-apoptotic properties of HDL particles in atherosclerosis is also increasingly strengthening [15], but there is a lack of such studies in the field of lymphoma.

Cysteine proteinase inhibitor, cystatin C (Cys C), is necessary for regulating intracellular and extracellular protein degradation. Previous research has demonstrated that Cys C is involved in altering the proteolytic system in cancer in such a way that elevated levels of plasma Cys C are associated with a poor prognosis in these patients [16] [17].

Additionally, regarding the inflammatory aspect, several studies suggested that CTSS might contribute to the inflammation process in various diseases, including cancer [4] [18].

The aim of the study was to analyse the association of CTSS with Cys C, HDL subclasses, cholesterol, and inflammatory markers in lymphoma patients in order to better understand the mechanisms of lymphomagenesis and lymphoma progression. Additionally, the objective was to evaluate the CTSS as a potential biomarker in lymphoma.

## Materials and methods

### Administrative study procedures

The study was conducted at the University Clinical Centre of the Republic of Srpska, Banja Luka, Bosnia and Herzegovina in accordance with the Declaration of Helsinki and was approved by the Ethics Committee of the University Clinical Centre of the Republic of Srpska, Banja Luka (No 01-19-51-2/20), and at the Faculty of Medicine at the University of Banja Luka (No 18/4.3.95/2020). Informed consent was obtained from all subjects involved in the study.

### Subjects

The study enrolled 55 newly diagnosed lymphoma patients between July 2020 and April 2022. Of the 55 patients, 11 patients had HL, 44 had NHL, and all 44 patients were B-cell NHL. Inclusion and exclusion criteria for the study group, along with those for the control group, as well as lymphoma classification, therapy, and monitoring of therapy success, are described in detail in a recently published paper by Mirjanic-Azaric et al. [18]. Only 25 patients were evaluated in the middle of treatment therapy (point of reassessment). Demographic and clinical data of the participants, such as age, sex, and clinical stage, were obtained from their medical history.

### Laboratory analyses

The patient’s blood samples were obtained before the 18F-2-fluoro-2-deoxy-D-glucose-positron 415 emission tomography/computed tomography (FDG-PET/CT), after overnight fasting (first time-point) and at the middle of the treatment immunochemotherapy (point of reassessment, second time-point) also after fasting, the same day of (and prior to) the FDG-PET/CT diagnostics. The blood was collected into one EDTA sample tube (for plasma) and one serum sample tube (for serum) before immediate centrifugation at 1500×g for 10 min at 4°C for plasma and 3000×g for 10 min for serum and then stored at -80°C until required.

CTSS levels were measured using the Invitrogen ELISA kit (Thermo Fisher Scientific, Inc., Waltham, MA, USA), following the manufacturer’s protocol. In brief, serum diluted 1:100 was added to microtiter plate wells pre-coated with the monoclonal antibody for CTSS capture. The minimum detectable dose of human CTSS was 4 pg/mL. The analysis of the standard curve and triplicate samples confirmed that the coefficient of variation (CV) was <10% for intra-assay and <12% for inter-assay, as instructed by the manufacturer.

The non-denaturing 3%–31% polyacrylamide gradient gel electrophoresis method was employed to separate plasma HDL particles [19].

Cys C was measured using the Cobas e 801 analyser (Roche Diagnostics GmbH, Mannheim, Germany), interleukin-6 (IL-6) and ferritin levels were assessed using the ADVIA Centaur XP system (Siemens Healthineers USA, United States). Total cholesterol (TC), LDL-C, HDL-C, LDH, and CRP were quantified using standard procedures on an Alinity Abbott analyser (Abbott Laboratories, IL, USA).

### FDG -PET/CT scan

Baseline and posttherapy FDG- PET/CT were performed on the same scanner (Discovery 610, GE Healthcare, Milwaukee, WI, USA). The response to therapy was evaluated using the Deauville five-point scale (Deauville criteria) [20]. Patients were categorised into three groups: complete metabolic responders (score 1 or 2), partial metabolic responders (score 3 or 4), and non-responders/progressive metabolic disease (score 5).

### Statistical analysis

Data are presented as mean ± standard deviation for normally distributed continuous variables, median with interquartile range for skewed data and relative and absolute frequencies for categorical variables. Continuous variables were compared between three groups using ANOVA with Tukey’s post hoc test for subgroup differences or Kruskal Wallis with Mann Whitney as a post hoc test. Analysis of covariance (ANCOVA) and Quade’s test were applied to investigate the influence of age and gender as confounders on the difference in normally distributed and skewed variables, respectively. Categorical variables were tested using the Chi-squared test. The correlations between the variables were estimated using Spearman’s correlation coefficient (r). Discriminative abilities of investigated parameters for lymphoma detection were assessed by receiver operating characteristic (ROC) curve analysis, and accuracy was presented as the area under the receiver operator characteristic curve (AUC) [21]. The statistical analyses were performed with PASW Statistics, v. 27, software (Chicago, Illinois, USA).

## Results

### Baseline patient characteristics and differences in examined parameters in the study

The median age of control subjects with an interquartile range was 49 (36–55), and they were significantly younger than NHL patients whose median age was 65 (55–72), P<0.001. The HL group consisted of the younger subjects [34 (24–71)], but their age was not significantly different from that of the previous groups. The distribution of genders was significantly different across the groups. Males were more prevalent in HL and NHL patient groups [6 (54.5%) and 22 (50.0%), respectively] than in controls [9 (25.7%), P=0.025]. The results showed that patients with HL and NHL were at similar disease stages at the baseline (Table 1). Among NHL subtypes, the follicular subtype of the disease was the most prevalent (47.73%), followed by diffuse large B-cell lymphoma (DLBCL) (31.81%) and small lymphocytic lymphoma (SLL) (9.10%) (Table 1).

**Table 1:** Main clinical characteristics of HL and NHL patients. HL, Hodgkin lymphoma; NHL, non-Hodgkin lymphoma; DLBCL, diffuse large B- cell lymphoma; SLL, small lymphocytic lymphoma; LPL, lymphoplasmacytic lymphoma; MCL, mantle cell lymphoma; MZL, marginal zone lymphoma; Compared by Chi square test.

	HL-patients, n=11	NHL-patients, n=44	P-value
Stage of cancer			
Stage I, n (%)	/	4 (9.09)	0.615
Stage II, n (%)	3 (27.27)	6 (13.63)	
Stage III, n (%)	2 (18.18)	10 (22.73)	
Stage IV, n (%)	6 (54.54)	24 (54.55)	
Subtype of NHL			
DLBCL, n (%)	/	14 (31.81)	/
Follicular, n (%)		21 (47.73)	
Burkitt, n (%)		1 (2.27)	
SLL, n (%)		4 (9.10)	
LPL, n (%)		1 (2.27)	
MCL, n (%)		2 (4.55)	
MZL, n (%)		1 (2.27)	

The Deauville score was analysed in 25 lymphoma patients before and after therapy (6 HL and 19 NHL patients). The most frequent Deauville scores were 1 (33.33%) and 5 (33.33%) in HL patients, and scores 1 (31.58%) and 3 (36.84%) in the HNL group. Of the 25 patients, 32% had a Deauville score of 1, a score of 2 had 4%, 28% had a score of 3, 16% had a score of 4, and 24% had a score of 5.

In Table 2, the laboratory parameters are examined. Levels of CTSS and CyS were significantly higher in NHL patients than in control subjects. As expected, LDH was significantly higher in both lymphoma groups than in controls.

**Table 2:** Comparison of serum biomarkers of healthy subjects and patients with HL and NHL. Compared by ANOVA or Kruskal-Wallis test, ^*^significantly different from the control group by Tukey test (p<0.05) or Mann-Whitney test; ^ⴕ^significantly different from HL group by Tukey test or Mann-Whitney test (p<0.05). Abbreviations: CTSS, cathepsin S; Cys C, cystatin C; LDH, lactate dehydrogenase; TC, total cholesterol; HDL-C, high-density lipoprotein cholesterol; LDL-C, low-density lipoprotein cholesterol; CRP, C-reactive protein; IL-6, interleukin-6.

	Controls<br>n=35	HL patients<br>n=11	NHL patients<br>n=44	P-value
CTSS (μg/L)	9.97<br>(8.44–10.99)	10.83<br>(9.23–12.40)	12.20^*^<br>(9.75–14.57)	0.002
Cys C (mg/L)	0.82<br>(0.74–0.90)	0.81<br>(0.74–1.13)	1.03^*^<br>(0.88–1.24)	<0.001
LDH (U/L)	118<br>(103–153)	195^*^<br>(137–218)	164^*^<br>(137–203)	<0.001
TC (mmol/L)	5.43±0.95	4.08±0.62^*^	5.12±1.25 ^ⴕ^	<0.001
HDL-C (mmol/L)	1.47±0.30	1.07±0.25^*^	1.29±0.39^*^	<0.001
LDL-C (mmol/L)	3.45±0.92	2.51±0.73^*^	3.37±1.00^ⴕ^	0.009
HDL 2b (%)	38.9±11.1	48.7± 10.6	39.8±13.2	0.062
HDL 2a (%)	21.2±7.7	22.3±4.5	20.3±4.2	0.564
HDL 2 (%)	60.1±14.4	71.0±15.1	60.1±14.3	0.356
HDL 3a (%)	15.2±5.5	12.9±2.5	14.1±4.0	0.300
HDL 3b (%)	10.6±4.5	7.0±3.3^*^	9.4±3.6	0.022
HDL 3c (%)	13.8±8.0	9.1±5.1	16.54±11.2	0.074
HDL 3 (%)	39.6±14.4	29.0±9.5	39.9±14.3	0.058
CRP (mg/L)	0.80<br>(0.40–2.80)	29.05^*^<br>(3.53–51.95)	2.70^*^<br>(0.85–5.85)	<0.001
Ferritin (μg/L)	27.0<br>(13.0–64.0)	227.0^*^<br>(150.5–394.5)	78.5^*ⴕ^<br>(29.0–238.0)	<0.001
IL-6 (ng/L)	1.1<br>(0.7–2.4)	6.8^*^<br>(2.1–14.0)	2.0^*^<br>(1.2–3.9)	<0.001

We additionally evaluated the difference in normally distributed parameters (TC, HDL-C and LDL-C) between healthy, HL and NHL subjects using ANCOVA, with age and gender included as covariates. Differences in TC, HDL-C and LDL-C between groups were not confounded by age and gender (P=0.004, P=0.012, and P=0.013, respectively). However, CTSS, Cys C, LDH, and inflammatory parameters were compared using Quade’s test, which had the same confounders. Age and gender did not influence a difference in CTSS (P=0.036), CyS C (P=0.041), LDH and inflammatory parameters (P<0.001) between groups.

In view of its heterogeneity, NHL patients were divided into the two most represented lymphoma subtypes in our research: follicular lymphomas and DLBCL. Accordingly, we compared follicularlymphoma and DLBCL patients with the control group, and the results are presented in Table 3. All significantly different parameters were higher in both groups compared to the control subjects. There was no significant difference for CTSS, Cys C and LDH between follicular and DLBCL.

**Table 3:** Markers significantly different between the control group and the group of patients with follicular lymphoma and DLBCL before therapy. Compared by ANOVA or Kruskall Wallis test, *significantly different from control group by Tukey test (p < 0.05) or Mann Whitney test; Abbreviations: DLBCL, diffuse large B- cell lymphoma, CTSS, cathepsin S; Cys C, cystatin C; LDH, lactate dehydrogenase.

	Controls<br>n=35	Follicular lymphoma<br>(indolent)<br>n=21	DLBCL<br>(aggressive)<br>n=14	P-value
CTSS (μg/L)	9.97<br>(8.44–10.99)	11.8*<br>(8.35–14.28)	13.64*<br>(11.85–15.58)	<0.001
Cys C (mg/L)	0.82<br>(0.74–0.90)	1.03*<br>(0.89–1.36)	1.03*<br>(0.88–1.19)	<0.001
LDH (U/L)	118<br>(103–153)	168*<br>(147–198)	154*<br>(122–190)	0.002

### The correlation of CTSS with a proportion of the HDL subclasses and inflammatory parameters in NHL patients

In HL patients, only a correlation between CTSS and Cys C was shown (r=0.685, P=0.014). In NHLpatients, serum CTSS showed a negative correlation with HDL-C and the HDL2b subclasses but a positive correlation with the proportion of the HDL3a, HDL3b, and the sum of the HDL3 subclasses (r=0.506, P<0.001; r=0.411, P=0.006; r=0.335, P=0.026, respectively) (Table 4). Regarding the inflammatory biomarkers, there was a positive correlation between CRP and CTSS.

**Table 4:** Significant correlations between CTSS and proportion of the HDL subclasses, HDL-C and CRP in NHL before therapy. r, Spearman’s correlation coefficient; P, level of significance. CTSS, cathepsin S; HDL high-density lipoprotein; HDL-C, high-density lipoprotein-cholesterol; CRP, C-reactive protein.

Laboratory<br>parameters	HDL2b<br>(%)<br>r<br>(P)	HDL3a<br>(%)<br>r<br>(P)	HDL3b<br>(%)<br>r<br>(P)	HDL3<br>(%)<br>r<br>(P)	HDL-C<br>(mmol/L)<br>r<br>(P)	CRP<br>(mg/L)<br>r<br>(P)
CTSS<br>(μg/L)	-0.328<br>(0.030)	0.506<br>(<0.001)	0.411<br>(0.006)	0.335<br>(0.026)	-0.424<br>(0.004)	0.351<br>(0.019)

### The results of diagnostic values of serum biomarkers in NHL patients

**Figure 1:**
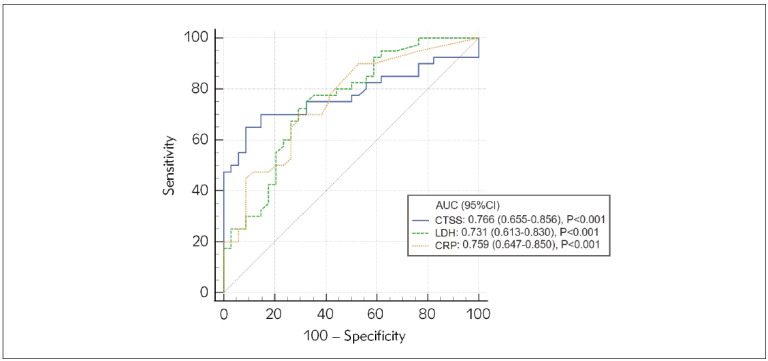
AUCs for CTSS in discrimination NHL patients from healthy subjects.<br>Abbreviations: AUC, the area under the ROC curve; CTSS, cathepsin S; LDH, lactate dehydrogenase; CRP, C-reactive protein

In further analyses, the receiver operating characteristic (ROC) curve was applied to investigate the accuracy of the analysed parameters in discriminating between NHL and control subjects. The highest discriminatory ability was found for CTSS with AUC=0.766, indicating a moderate diagnostic value for the detection of NHL. The optimal cut-off value for CTSS was 10.56 μg/L with a sensitivity and specificity of 73% and 74%, respectively. Figure 1 presented only parameters with an accuracy higher than 0.7.

### The changes in CTSS between two follow-up points in patients with lymphoma

**Figure 2:**
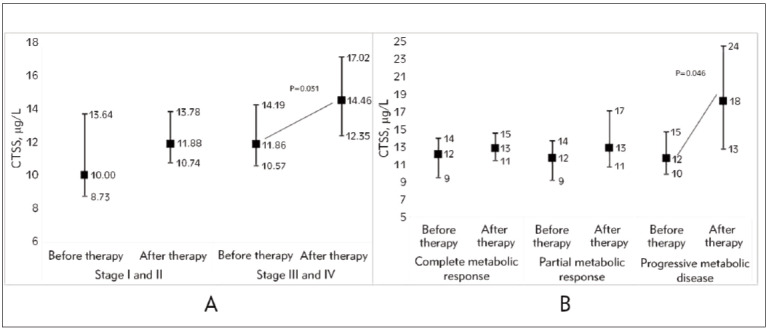
CTSS changes after therapy.<br>Figure 2 A, according to disease stages; Figure 2 B according to response to therapy Abbreviation: CTSS, cathepsin S.

Figure 2A shows significant changes in the CTSS after therapy in patients divided according to disease stages. CTSS levels significantly increased by approximately 12% in patients with disease stages III and IV. Additionally, patients were divided according to response to therapy (Figure 2B). Patients with progressive metabolic disease (Deauville score 5, n=6) had increased CTSS levels by 25% after therapy. After therapy in patients with partial metabolic response (n=11, Deauville scores 3 and 4) and patients with complete metabolic response on therapy (n=8, Deauville scores 1 and 2), CTSS changes were without statistical significance.

## Discussion

The presented results of the study showed that the serum CTSS and Cys C levels were significantly elevated in NHL patients compared to healthy subjects. These results are concordant with similar cross-sectional studies, which suggested that CTSS may have several potential implications in the development and progression of carcinoma [22] [23] [24].

Increased values of CTSS in NHL could lead to an impaired apoptosis control mechanism andincreased cell survival. Furthermore, extensive research has indicated that CTSS can be a therapeutic target [8], as supported by the results of our study. The elevated CTSS levels could be elucidated by a mechanism suggested by Dheilli et al., [25] namely, cancer cells interact with immune cells called CD4+ T-cells when CTSS is active, which helps tumours to grow. At the same time, they maintain distance from CD8+ T-cells, which would attack and kill cancer cells. Contrary to a study by Ma et al. [26], we did not observe a high level of CTSS in the HL group. This finding may result from different tumour micro-environments in HL characterised by a minority of neoplastic cells and an extensive inflammatory milieu [27]. Also, NHL generally involves a wider range of cells in the lymphatic system compared to HL and CTSS derived from tumour cells, and cells within the tumour microenvironment contribute to tumor-igenesis.

The role of Cys C has been proposed in relation to the modification of the proteolytic system in cancer. The findings that Cys C levels were higher in NHL patients than in healthy subjects are in accordance with previous studies [16] [28] [29]. Cystatins effectively inhibit a small amount of catalytically active proteases. Therefore, it may be assumed that the disturbance of signalling pathways, as seen in cancer, probably disables Cys C from inhibiting large amounts of CTSS, and that is probably why there was not a correlation between these two biomarkers.

When considering lipid parameters, only LDL-C values were notably lower in HL patients than in the control group. During an inflammatory state, also increased levels of reactive oxygen species can lead to the oxidation of LDL into oxidised LDL (ox-LDL) and a decrease in circulating LDL because oxidised LDL is taken up by macrophages at the site of inflammation. Furthermore, this is likely due to significantly elevated IL-6 values within this group of patients; it is widely recognised that cytokines can contribute to dyslipidemia [30]. Additionally, *in vitro* studies demonstrated the increased uptake of cholesterol from plasma by malignant cells to meet their own proliferation [31].

Low levels of HDL-C in haematological cancers indicate that HDL particles may play a crucial role in maintaining strict control over the proliferation and homeostasis of the hematopoietic system, potentially impeding malignant transformation [9] [10] [30]. In this study, significantly lower values of HDL-C were found in both types of lymphoma. These results are similar to those obtained in the study by Pedersen et al., [32], which showed that patients with low HDL-C have an increased risk for haematological cancers. The Spearman correlation analysis showed that circulating CTSS was negatively correlated with HDL-C in NHL. Increased cell proliferation and reduced apoptosis in lymphoma, which could be associated with elevated levels of CTSS, have led to higher cholesterol consumption and, consequently, reduced circulating HDL-C levels. This is also an explanation for the negative correlation of CTSS with HDL2b, considering that the HDL2 subclasses are significantly richer in cholesterol compared to the HDL3 subclasses. In NHL patients, we revealed a positive association of CTSS with HDL3a, HDL3b, and HDL3 lipoprotein particles. HDL3 subclasses are small, dense, protein-rich subclasses that exhibit pronounced anti-apoptotic, anti-oxidative, and anti-inflammatory properties [33] [34]. Molecules with anti-apoptotic effects can block specific enzymes or proteins in apoptotic signalling pathways, thereby disrupting or preventing program med cell death. The strong connection between CTSS and these particles could be attributed precisely to the anti-apoptotic characteristics of these subclasses. As far as we know, this is the first study that has shown an association between CTSS and the HDL3a and HDL3b subclasses, and the reduced apoptosis present in cancer could also be a consequence of this association. Moreover, the first demonstrated a positive correlation between CTSS levels and the proportion of anti-apoptotic subclasses HDL3a and HDL3b, which will enhance the understanding of disease mechanisms in NHL, potentially contributing to the development of new therapeutic strategies for this cancer.

This study supports the hypothesis that inflammation contributes to the development of cancer [35] [36] [37]. Contrary to the study conducted by Preti et al. [35], IL-6 did not prove to have diagnostic potential in patients with NHL.

Finding new and non-invasive biomarkers for early cancer detection has become crucial nowadays. ROC curve analysis demonstrated that CTSS can serve as a diagnostic marker for NHL. The AUC value for CTSS was higher than that of LDH, although LDH is still considered a valuable marker in the diagnosis and monitoring of NHL therapy.

This study demonstrates that the progression of cancer to later stages (stages III and IV) is associated with higher levels of serum CTSS compared to the early stages (stages I and II). Furthermore, the rise of CTSS levels after therapy with a Deauville score of 5 indicated that CTSS may be important in monitoring the success of lymphoma treatment. Observing lower levels of CTSS in patients with Deauville score 1 or 2, we emphasise that CTSS can indicate disease remission status.

It is important to note that this study has a relatively small number of patients. Due to vis major and unforeseen consequences of the COVID-19 pandemic on travelling restrictions and diagnostics availability, a significant number of our patients were unable to return for a reassessment of FDG-PET/CT after treatment. We consider this to be a significant limitation of our research. Based on these findings, further research will be customised to specific patient groups, taking into account distinctions in lymphoma type, severity, and prognosis. We strongly believe that some other studies with a larger number of patients will confirm these results.

## Conclusions

CTSS is significantly elevated in patients with NHL but not in HL and has the potential to be a newdiagnostic biomarker for the detection of NHL. New biomarkers are significant not only for diagnosis but also for their ability to uncover new avenues for therapeutic intervention. Additionally, this study was the first to unveil the association between CTSS levels and the proportions of HDL3a and HDL3b subclasses in NHL patients, which could play a pivotal role in the enhanced survival of cancer cells. Further studies with more patients are needed to confirm the diagnostic potential of serum CTSS in NHL.

## Dodatak

### List of abbreviations

HL, Hodgkin lymphoma;<br>NHL, non-Hodgkin lymphoma;<br>DLBCL, diffuse large B-cell lymphoma;<br>SLL, small lymphocytic lymphoma;<br>LPL, lymphoplasmacytic lymphoma;<br>MCL, mantle cell lymphoma;<br>MZL, marginal zone lymphoma;<br>CTSS, cathepsin S;<br>Cys C, cystatin C;<br>LDH, lactate dehydrogenase;<br>TC, total cholesterol;<br>HDL-C, high-density lipoprotein cholesterol;<br>LDL-C, low-density lipoprotein cholesterol;<br>CRP, C-reactive protein;<br>IL-6, interleukin-6;<br>AUC, area under the receiver operating characteristic;<br>ROC, receiver operating characteristic.

### Funding

This work is supported by the Ministry for Scientific/Technological Development, Higher Education and Information Society, Government of the Republic of Srpska (Grant. No. 1257023).

### Conflict of interest statement

All the authors declare that they have no conflict of interest in this work.
